# Integrating environmental health and genomics research in Africa: challenges and opportunities identified during a Human Heredity and Health in Africa (H3Africa) Consortium workshop

**DOI:** 10.12688/aasopenres.12983.1

**Published:** 2019-08-27

**Authors:** Bonnie R. Joubert, Kiros Berhane, Jonathan Chevrier, Gwen Collman, Brenda Eskenazi, Julius Fobil, Cathrine Hoyo, Chandy C. John, Abera Kumie, Mark Nicol, Michèle Ramsay, Joshua Smith, Adrie Steyn, Desire Tshala-Katumbay, Kimberly McAllister

**Affiliations:** 1 National Institute of Environmental Health Sciences, National Institutes of Health, Durham, NC, USA; 2 Keck School of Medicine, University of Southern California, Los Angeles, CA, USA; 3 McGill University, Montreal, Canada; 4 University of California, Berkeley, San Fracisco, CA, USA; 5 University of Ghana, Accra, Ghana; 6 North Carolina State University, Raleigh, NC, USA; 7 Indiana University School of Medicine, Indianapolis, IN, USA; 8 Addis Abada University, Addis Abada, Ethiopia; 9 University of Cape Town, Cape Town, South Africa; 10 Sydney Brenner Institute for Molecular Bioscience, Faculty of Health Sciences, University of the Witwatersrand, Johannesburg, South Africa; 11 Johns Hopkins University, Baltimore, MD, USA; 12 Africa Health Research Institute, Durban, South Africa; 13 Oregon Health & Science University, Portland, OR, USA

**Keywords:** H3Africa, G x E, environmental health, global environmental health, Africa, workshop, gene-environment interactions

## Abstract

Individuals with African ancestry have extensive genomic diversity but have been underrepresented in genomic research. There is also extensive global diversity in the exposome (the totality of human environmental exposures from conception onwards) which should be considered for integrative genomic and environmental health research in Africa. To address current research gaps, we organized a workshop on environmental health research in Africa in conjunction with the H3Africa Consortium and the African Society of Human Genetics meetings in Kigali, Rwanda. The workshop was open to all researchers with an interest in environmental health in Africa and involved presentations from experts within and outside of the Consortium. This workshop highlighted innovative research occurring on the African continent related to environmental health and the interplay between the environment and the human genome. Stories of success, challenges, and collaborative opportunities were discussed through presentations, breakout sessions, poster presentations, and a panel discussion. The workshop informed participants about environmental risk factors that can be incorporated into current or future epidemiology studies and addressed research design considerations, biospecimen collection and storage, biomarkers for measuring chemical exposures, laboratory strategies, and statistical methodologies. Inclusion of environmental exposure measurements with genomic data, including but not limited to H3Africa projects, can offer a strong platform for building gene-environment (G x E) research in Africa. Opportunities to leverage existing resources and add environmental exposure data for ongoing and planned studies were discussed. Future directions include expanding the measurement of both genomic and exposomic risk factors and incorporating sophisticated statistical approaches for analyzing high dimensional G x E data. A better understanding of how environmental and genomic factors interact with nutrition and infection is also needed. Considering that the environment represents many modifiable risk factors, these research findings can inform intervention and prevention efforts towards improving global health.

## Background

The Human Heredity and Health in Africa (H3Africa) initiative began as a joint effort of the National Institutes of Health (NIH) Common Fund Global Health Program and the Wellcome Trust, in partnership with the African Society of Human Genetics. H3Africa aims to support the establishment of a sustainable African research infrastructure for the study of the genetic and environmental contributors to disease and health ^1^. The work of this Consortium includes common diseases such as cardiovascular ^2^, neurological ^3^, respiratory ^4^, kidney ^5^, and other non-communicable diseases, as well as research in infectious diseases. Developments in pharmacogenomics ^6^ and the human microbiome ^7^ are underway, and many studies incorporate information about HIV, malaria, and tuberculosis. The Consortium also promotes opportunities for training in bioinformatics ^8– 10^, supports three biorepositories on the African continent, and facilitates policy development and guidelines with recommendations on ethical issues ^11– 13^.

Much of the work within the first phase of H3Africa (August 2012 – July 2017) focused on building genomic infrastructure, research networks, and training. Phase two of the program began in 2017 and involved co-funding from multiple NIH institutes, including the National Institute of Environmental Health Sciences (NIEHS), to include more coverage of environmental exposures in H3Africa projects. Several projects began to incorporate environmental exposures, but existing data, planned data, or suitable specimen collection around such exposures were limited in scope. A need was identified to incorporate more education, training, and research on environmental health or the exposome ^14– 16^ within the H3Africa network. This enhancement would enable greater ability of ongoing H3Africa projects to evaluate both genomic, epigenomic, and environmental risk factors of disease in Africa. To support this effort, NIEHS facilitated a one-day workshop on environmental health entitled, “Environmental Health in Africa: Opportunities to Expand Research Capacity in the H3Africa Consortium.” The workshop was held one day prior to the 12th H3Africa Consortium meeting in Kigali, Rwanda, in September 2018. A total of seven speakers and five panelists were invited to lead the workshop, representing environmental health research expertise in the African setting. Scholarships enabled travel support for African-based pre- and post-doctoral trainees who presented posters. The workshop agenda, speaker biographies, poster abstracts, bibliography of recommended reading, and participant list can be found in the workshop meeting book posted on the meeting website.

## Objectives

The workshop was opened by Dr. Gwen Collman, Director of the NIEHS Division of Extramural Research and Training. Dr. Collman described the long history of NIEHS-supported environmental health research in Africa and the unique exposures and health challenges. She described the commitment of NIEHS to global environmental health and involvement in the H3Africa consortium. Dr. Kim McAllister, Program Administrator at NIEHS, provided brief remarks on the charge of the workshop, on behalf of the NIEHS organizing team. She highlighted the opportunity for H3Africa projects to expand exploration for both genomic and environmental risk factors of complex diseases in Africa. The objectives of the workshop were to highlight current and innovative research occurring on the African continent related to the interplay between the environment and the human genome. The workshop was designed to enable face to face interactions between Consortium members and environmental health experts to facilitate collaborative opportunities and global networking. Sharing of expertise, research recommendations for study design, biospecimen collection and storage practices, and statistical methodologies were discussed, as well as the current research challenges and needs of existing H3Africa projects. **

## Survey of environmental data and interests in H3Africa projects

**Figure 1.**
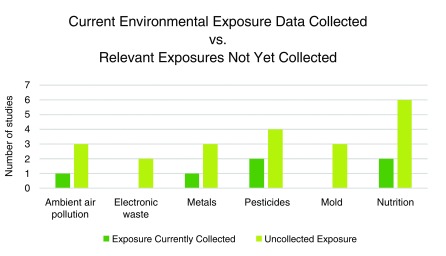
Survey results on environmental exposures and interests of H3Africa projects. Nine projects provided responses to the questions, “What environmental exposure data are you currently collecting in your study population?” and “What environmental exposure(s) pose a public health issue for your study population that you are not already collecting?”

**Figure 2.**
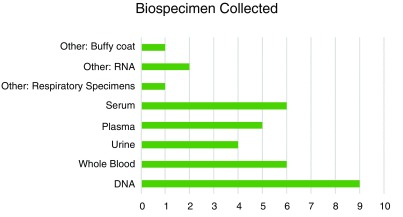
Biospecimens collected across nine H3Africa projects. Respiratory specimens indicate samples from the respiratory tract.

To better understand the data and interests of existing H3Africa projects, a short survey including fifteen questions was sent to all H3Africa project PIs by the coordinating center, to collect information on environmental exposures in H3Africa projects. Data collected on questionnaires, biomarkers of environmental exposures, monitoring data, or related information was reported. Characteristics of the H3Africa project study populations were collected including life stage, location, important co-morbidities, key health outcomes, and current environmental data. The survey also asked about interests in collecting more environmental exposure data in existing study populations. Nine projects completed the survey prior to the workshop ( Figure 1; Table 1). The study populations of these projects represented West Africa (40%), Central Africa (6.7%), Eastern Africa (26.7%), and Southern Africa (26.7%). (There were no study populations from Northern Africa represented by projects completing the survey). The primary outcomes of interest from these projects were cardiovascular disease, infectious disease, and psychosocial or mental health disorders. DNA was the most commonly reported biospecimen collected and stored ( Figure 2), and approximately 55% of respondents stated that additional biospecimen collection was not possible for their study population(s). This indicated some limitations in the scope of environmental data that can be added using biospecimens (for example, biomarkers measured in urine samples).

**Table 1.** Summary of pre-workshop survey on environmental health research in existing H3Africa projects ^†^.

Project location (s)	Collected environmental exposure data	Data of interest but not yet collected	Questionnaires capturing environmental exposures	Biospecimens collected	Additional biospecimen collection	ELSI ^‡^ questions	Training needs	Issues for sample management
South Africa			No	DNA	No	No		
East Africa; West Africa; South Africa	Indoor air pollution; nutrition	Nutrition	No	Whole blood; DNA; Urine; Plasma; Serum	No			Laboratory training; Communication strategies with project sites; Storage capacity or related storage issues
Central Africa; East Africa		Indoor air pollution; Nutrition	No	Whole blood; DNA	No	Don't know	Laboratory training; Bioinformatic training; Statistical analysis skills development	Storage capacity or related storage issues
West Africa; South Africa	Indoor air pollution	Ambient air pollution; Metals; Pesticides; Mold; Nutrition	Yes	Whole blood; DNA; Urine; Plasma; Serum; Other: RNA, respiratory specimens (swabs, sputum)	Yes	Yes	Laboratory training; Bioinformatic training; Statistical analysis skills development	Laboratory training; Storage capacity or related storage issues; Other: timing of specimen collection
West Africa	Indoor air pollution; Metals; Pesticides	Ambient air pollution; Indoor Air Pollution; Electronic Waste; Metals; Pesticides; Mold; Nutrition	No	DNA; Other: RNA	Yes	Yes	Laboratory training; Bioinformatic training; Statistical analysis skills development	Laboratory training; Storage capacity or related storage issues
West Africa	Ambient air pollution	Indoor air pollution; Electronic waste; Metals; Pesticides; Mold; Nutrition	Yes	Whole blood; DNA; Urine; Plasma; Serum	Yes	Don't know		Storage capacity or related storage issues
East Africa			No	Whole blood; DNA; Serum	Yes	Don't know	Bioinformatic training	Laboratory training
East Africa; West Africa; South Africa	Indoor air pollution; Pesticides; Nutrition	Indoor air pollution; Pesticides; Nutrition	Yes	DNA; Urine; Plasma; Serum	Yes	No	Laboratory training; statistical analysis skills development	Storage capacity or related storage issues; Funding
West Africa	Indoor air pollution	Ambient air pollution	Yes	Whole blood; DNA; Plasma; Serum; Buffy Coat	Yes	No	Laboratory training; Bioinformatic training; Statistical analysis skills development	Laboratory training; Communication strategies with project sites; Storage capacity or related storage issues

^†^ A survey to better understand environmental exposure data collected and of interest for H3Africa projects was distributed to all consortium projects prior to the workshop by the H3Africa coordinating center. Responses were available for nine projects to the following questions:

Project location (s): Geographic location(s) of study population(s)

Collected environmental exposure data: What environmental exposure data are you currently collecting in your study population? ( Figure 1 data)

Data of interest but not yet collected: What environmental exposure(s) pose a public health issue for your study population that you are not already collecting? ( Figure 1 data)

Questionnaires capturing environmental exposures: Do you capture information about environmental exposures in your study questionnaires?

Biospecimens collected: What samples are collected and available for research from your study participants? ( Figure 2 data)

Additional biospecimen collection: Is additional biospecimen collection possible for your study population (e.g. at follow-up visits with informed consent)?

ELSI questions: Do you have questions about ELSI issues for collecting environmental data (e.g. information about environmental exposures not specifically described in informed consent)?

Training needs: What are the training needs needed by your project to strengthen environmental health research capacity?

Issues for sample management: What are the critical issues for storing/managing samples for environmental analysis?

Two responses were also provided to the question, “What else may be a major challenge or obstacle for expanding environmental measures or environmental health research capacity in your project(s)?” Responses: 1) Logistics of sample collection and 2) Funding for additional exposure measurements, understanding relevance of additional exposures to outcomes being measured.

^‡^ ELSI: Ethical, legal, and social implications.

The study respondents reported needs for training in the areas of laboratory methods, bioinformatics, and statistical analysis of complex environmental exposures. The most common critical issues reported to enable more environmental health research were storage capacity and laboratory training. Improvements in the communication strategies with project sites, timing of specimen collection, better implementation of sample collection, and a better understanding of the relevance of additional exposures to measured outcomes were also noted. Overall, the surveys indicated substantial interest in learning more about environmental exposures and risk assessments in the H3Africa study populations, but limited data was currently in hand or planned for collection. Training and education for junior investigators and partnerships with expert laboratories outside of Africa were emphasized.

## Presentations

The workshop presenters covered experimental design, exposures of interest, exposure measurements, biospecimen collection and storage, and laboratory and statistical methodologies. They also discussed training needs and other challenges, populations at risk, health outcomes of interest for represented populations, and collaborative opportunities. The scientific content of the presentations is organized here by exposure category.

### Electronic waste

The first presenter in the workshop was Dr. Julius Fobil from the University of Ghana, one of the principal investigators of the Michigan-West Africa Global Environmental and Occupational Health ( GEOHealth) Hub. He described the environmental exposures associated with informal e-waste recycling practices in West Africa (particularly in Ghana at the Agbogbloshie dumpsite and a comparison site at Madina-Zongon, 10 km north). E-waste workers and neighboring communities are exposed to multiple toxic exposures related to recycling activities (such as open burning and manual dismantling) of electronics in a highly concentrated area ^17^. High levels of toxic metals (especially lead) and persistent organic pollutants have been found in air, water, soil, and food samples on and near e-waste sites that far exceed United States Environmental Protection Agency (U.S. EPA) and World Health Organization (WHO) thresholds of safe exposure. The GEOHealth Hub project goals include: characterization of specific exposures derived from electronic waste recycling activities, assessment of biological markers of dose of exposure, and evaluation of health outcomes (particularly respiratory, morbidity, and cancer risks but also cardiovascular and musculoskeletal outcomes) associated with these exposures. Biological samples (blood and urine) were used to measure metals and flame retardants, polycyclic aromatic hydrocarbons (PAHs), and other organic compounds, and ambient and personal air monitoring were used to measure air pollution. Interactions between occupational exposures and other environmental exposures were also assessed through a detailed questionnaire. While the hub’s activities aim broadly to develop new methods for assessing exposure in the informal sector in the hope of developing effective and efficient interventions that reduce the exposures, ongoing analyses have been expanded to include investigation of associations between exposures in the e-waste dismantling industry and biologic effects as measured by the following molecular biology platforms: i) gene-expression profiling of epithelial cells from the oral cavity and nasal turbinate; ii) metabolomic profiling of plasma; and iii) methylation profiling of blood DNA. Preliminary results have found exceptionally high PM2.5 exposures for e-waste workers using personal air monitoring filters. Discussions following this presentation focused on the complexities of impacting better e-waste worker protections and informing government and city policies to improve workers’ lives. The investigators hope to explore pilot interventions to reduce exposures at the Agbogbloshie site in Ghana and scale-up studies in other West Africa countries in the future.

### Ambient air pollution

Dr. Abera Kumie, from Addis Ababa University, presented recent findings from the Eastern Africa GEOHealth Hub studying ambient air pollution exposure and child respiratory health in Ethiopia, Uganda, Rwanda, and Kenya. Air pollution is an important global challenge resulting in 4.3 million deaths from indoor air pollution and 3.7 million deaths from outdoor/ambient air pollution each year (WHO, 2017). Air pollution poses a heavy burden in Eastern Africa, and Dr. Kumie noted exposure statistics for Ethiopia and sources of exposure, with a primary focus on PM2.5. Sources of air pollution were both mobile and stationary, with power generation, factories, and household biomass contributing significantly to substantial burdens of pollutants CO, NO _2_, O _3_, SO _2_, and other particles. Some of the strongest associations with adverse health outcomes have been observed globally for PM 2.5, and in Africa the levels of PM2.5 and other air pollutants are much higher than WHO limits. Dr. Kumie noted a publication from the *New England Journal of Medicine* in 2013 reporting global disability-adjusted life years (DALYs) attributed to household air pollution from solid particles and exposure to ambient particulate matter pollution ranking within the top 10 leading risk factors for DALY in 1990 and 2010 ^18^. In the Eastern Africa GEOHealth Hub, air pollution exposure (mainly PM2.5 and also CO in a subsample of households) was measured through air quality monitoring (using central site monitor, sub-city monitor, and home-based monitors), questionnaires (using residential history, time activity logs, and housing characteristics), and personal exposure monitoring (using Particle and Temperature Sensors [PATS] + Ultrasonic Personal Aerosol Samplers [UPAS] in homes for PM2.5 and MA200 for black carbon monitoring) to assign household level exposures. Pictures and brief explanations of the technologies used for air pollution monitoring to measure PM2.5 were also shared (e.g., E-sampler, BAM-1022, and PATS + UPAS MA200). More details of the GEOHealth Hub study populations were described, including study sites, study protocols and staff training, and data was placed in context with the overall hub aims. Preliminary data such as descriptive statistics of the study populations were also shared. Overall, this presentation described the public health issue of air pollution in Eastern Africa, provided examples of measuring ambient air pollution in an African setting, and shared equipment and methodology recommendations with the workshop participants.

### Mycotoxin

Dr. Joshua Smith, a post-doctoral fellow in Dr. John Groopman’s laboratory at Johns Hopkins University presented ongoing work associated with the translation of basic research to public health prevention related to mycotoxin exposures, which could serve as a model for other exposures. Mycotoxins, including aflatoxins, are secondary metabolites of fungi that are frequent contaminants of improperly stored food crops and are highly carcinogenic to the liver (and associated with increased liver cancer risk). Extensive mechanistic research over many years has enabled the identification of an aflatoxin B1-lysine adduct in serum using isotope-dilution liquid chromatography/tandem mass spectrometry as a highly effective biomarker for aflatoxin exposure. Multiple primary and secondary prevention mitigation strategies have been used to both reduce aflatoxin exposure with improved food storage methods and dietary changes and introduce chemopreventive interventions using broccoli sprouts, green tea, chlorophyllin, and other agents to induce hepatic detoxification and reduce aflatoxin absorption. Primary prevention strategies addressing the reduction of aflatoxin contamination of staple groundnut foods have been applied in Guinea, and these have successfully reduced aflatoxin exposure ^19^. These have included altering many postharvest processing practices for the groundnuts, including hand sorting of the nuts, sun drying nuts on natural fiber mats, storing food in natural fiber bags (rather than plastic or synthetic bags), and using insecticides in the storage facilities. Aflatoxin exposure is also associated with stunting (reduced growth in young children) and ongoing clinical trials (including a trial funded by the Bill and Melinda Gates Foundation in Malawi) are exploring the seasonal effects of aflatoxin exposure on children’s growth and development.

### Heavy metals

Dr. Cathrine Hoyo from North Carolina State University presented on heavy metal exposure and cardiometabolic phenotypes. She shared results from her work in the Newborn Epigenetic Study (NEST) evaluating metals exposures and obesity in just over 310 children living in North Carolina, USA. Exposures discussed included cadmium and lead, which was found in the blood samples of pregnant women during the first trimester. Women with elevated cadmium and lead exposure were geographically clustered based on prenatal mailing addresses. This clustering in space of women with elevated levels of cadmium and lead was followed by sampling and measuring soil, house dust, and water to identify potential sources and routes of exposure and guide prevention efforts ^20^. Although no cadmium or lead was found in water, these metals contaminated soils and house dust of women with elevated concentrations suggesting soil tracked indoors and part of house dust could represent an important source of cadmium and lead exposures. Concentrations of these metals were also higher on hand wipes of this geographic cluster, suggesting that inadvertent ingestion of contaminated house dust could be a potential route of exposure.

Cadmium and lead are in the top ten chemicals of concern by the International Agency for Research on Cancer ( IARC) and Agency for Toxic Substances and Disease Registry ( ATSDR), because: their industrial applications are numerous, they are poorly excreted (hence the heavy metal burden increases with increasing age), and they sizably contribute to cardiovascular and metabolic disease, which are diseases in the top ten global causes of mortality. While smoking is a major source of cadmium, it is estimated that non-smokers also ingest ^~^1µg per day from diet as plants take up this compound from the air, contaminated soil, and water ^21^. The European Union estimates that cadmium exposure will increase with the projected increase in the production of batteries for electric vehicles, for which cadmium is an important component ^21^. The yet unexplained higher propensity for exposure among women will affect unborn children and their offspring for generations to come as observed with juvenile obesity ^22^. In addition to key highlights to her research on the health effects of metals in children, Dr. Hoyo described considerations for biosample collection for investigators interested in studying metals. Notably, she described recent advances in Inductively Coupled Plasma-Mass Spectrometry that can be leveraged to identify multiple metals in limited specimens. She also described contamination issues that may arise when collecting samples, and methods to help obtain good data for these exposures.

### Nutrition and toxicodietary exposures

Dr. Desire Tshala-Katumbay from Oregon Health and Science University discussed nutrition and toxicodietary exposures in Africa. He noted a broad goal to develop strategies to reduce the burden of neurotoxicity associated with cassava intake, a staple food for more than 600 million people around the world. He discussed the relationship between food (cassava), cyanogenic toxicants, and outbreaks of a motor system disease, known as konzo, in sub-Saharan Africa. The causes of konzo have largely remained unclear, and Dr. Tshala-Katumbay’s research is focusing on increasing the understanding of lifespan interactions between environmental stressors (toxicants), diet (nutritional status), and genetics in relation to motor system degeneration. These studies are exploring the biomarkers and toxicity mechanisms of cassava-associated motor/cognition deficits, testing a central hypothesis that genetic variations, along with epigenetics and microbiome variation, modulate risks for cassava-associated neurodegeneration. A key premise to these studies is that production of cyanate, a metabolite of cyanide (internally produced upon ingestion of cassava cyanogens), carbamylates lysine-enriched proteins to induce conformational changes and alters vital molecular functions including, possibly, transcription and cell signaling. Dr. Tshala-Katumbay’s project encompasses many scientific fields including plant-microbiota-host interactions, functional metagenomics, transgenerational epigenetics, and neuropathogenic patterns. He emphasized ongoing capacity building through the training and mentoring of young investigators in the Democratic Republic of the Congo through active participation in supervised field work, training in ethics and responsible conduct for research, field epidemiology, neurology, nutritional toxicology, and environmental health science.

### Pesticides

Dr. Jonathan Chevrier, from McGill University, presented on Indoor Residual Spraying (IRS) for Malaria Control and Child Development in the South African Venda Health Examination of Mothers Babies and their Environment (VHEMBE) birth cohort study located in the Vhembe district of Limpopo, South Africa. According to the World Malaria Report in 2018, IRS is used in 84 countries around the world, exposing more than 100 million people, primarily those living in Africa ^23^. Chemicals used in IRS include DDT (dichlorodiphenyltrichloroethane) and pyrethroids, which may have endocrine disrupting effects in rodents and humans. Although IRS is effective in reducing mortality due to malaria, the potentially negative side effects of IRS have not been fully evaluated. Dr Chevrier described the interesting history of pyrethroids including their source and initial development in the early 1800s, the active structure identification in 1920, the generation of the synthetic product in 1960-70, and widespread generation for use in the insecticide market beginning in 1986. Pyrethroids are projected to represent a $6 billion industry by 2021. Distribution of pyrethroids use for IRS around the world was also described in detail, including the WHO-reported use of alpha-Cypermethrin, Deltamethrin, lambda-Cyhalothrin, and Cyfluthrin in 2015. The history of DDT was also briefly summarized, beginning with the initial discovery in 1939, recognition for Paul Müller’s DDT discovery in 1948 (Nobel Prize), the 1962 publication of the book *Silent Spring* by Rachel Carson warning of harmful effects to DDT-exposed wildlife, the 1970s banning of DDT in Western Countries, the 2001 Stockholm Convention on Persistent Organic Pollutants, and the 2006 WHO recommendation of IRS upscaling to combat malaria. Persistence, distribution in the body, excretion, and related characteristics of DDT and pyrethroids and their metabolites and known toxicities were presented.

Dr. Chevrier provided recommendations on starting points for studies interested in assessing exposure to pyrethroids and DDT/DDE (dichlorodiphenyldichloroethylene) and finally, provided an example of his and his colleague’s work in the VHEMBE cohort. He reported that IRS was associated with elevated serum DDT/DDE and urinary pyrethroid metabolite concentrations in peripartum samples collected from 751 VHEMBE women ^24, 25^. Associations between maternal exposure to DDT and hypertensive disorders of pregnancy ^26^, larger birth size ^27^, and higher body composition measures, and marginally altered motor development were found among VHEMBE girls at age 1 and/or 2 years ^28^. Prenatal exposure to pyrethroids was also associated with lower body composition measures among boys ^28^ and altered motor development among girls aged 1 to 3.5 years ^29, 30^. Given these results and the widespread exposure to these insecticides in African populations, the VHEMBE study has also provided education and outreach to many of the participants to reduce exposure.

### Biomarkers of exposure, inflammation, and infectious diseases

Dr. Chandy John from Indiana University School of Medicine described the possible association of trace elements with risk of severe malaria and neurodevelopmental impairment in Kenyan children. Past research has suggested that trace element deficiencies have been associated with increased infection and may also affect cognition. Dr. John discussed the use of various biospecimens (including blood, plasma, serum, urine, and hair) and testing methods for exploring exposures in humans. Whole blood is recommended for providing best blood level measures and hair is useful for evaluation of long-term exposures. Dr. John measured trace metal levels in children during episodes of severe malaria (cerebral malaria and severe malarial anemia), comparing them to levels for children without malaria to assess the relationship of trace metal levels to risk of severe malaria and cognitive impairment. Dr. John also mentioned the potential ethical, legal, and social issues involved in toxic levels or severe deficiencies of trace elements because of the obligation to report such findings to study participants and because of the political implications of tracing the sources of toxic exposures. The project utilized the NIEHS Children’s Health Exposure Analysis Resource (CHEAR) ^31, 32^ to measure 17 trace elements in whole blood samples using Inductively Coupled Plasma Mass Spectrometry as the gold standard method for trace element measurements. No severe deficiencies or toxic levels of trace elements were found. However, the preliminary results suggest a significant association between numerous altered trace element concentrations (including lower magnesium, molybdenum, selenium, and zinc levels and increased levels of copper and tin) with cerebral malaria and severe malarial anemia (unpublished study).

## Breakout sessions

Participants gathered in smaller groups in the afternoon to discuss common and emerging environmental exposures of interest to H3Africa researchers. Breakout group discussions were focused on exposures of interest including: electronic waste, ambient and indoor air pollution (including tobacco smoke), metals and mycotoxins, pesticides and nutrition/toxicodietary exposures, and biomarkers of chemical exposures and infectious diseases. Each group was asked to comment on the important health outcomes associated with the exposure category, what could be done now to measure exposures of interest in existing studies, example research they may be involved in, training needs to build environmental health research capacity, critical issues for biorepositories storing/managing samples for environmental analysis, and ethical, legal, and social implications. General themes of interest across the breakout group discussions were: identifying what exposures should be measured when studying specific health outcomes, clinical training to recognize symptoms related to hazardous environmental exposures, access to existing data/records/biological samples to assess exposures, use of questionnaires to determine suitability of existing samples for biomarkers of environmental exposures, and biobanking of samples. Key points and discussion from select breakout sessions are provided below.

### Ambient and indoor air pollution (including tobacco smoke)

Ambient air pollution was agreed to be a significant hazard in urban areas in Africa, whereas indoor pollution, including indoor cooking, constitutes a problematic exposure in rural areas and slums in large cities. Different cultures and practices across Africa can make comparisons complex and likely require a multidisciplinary approach. Measurements of exposures need to be affordable, robust, validated across different settings, and coordinated through a harmonized approach for data generation and storage. Some African countries monitor and document air pollution, albeit to different levels of complexity and geographic coverage, whereas others have no resources to do so. Despite rare pockets of expertise and activity, there was generally a sense that there was a lack of political will in most African countries to divert resources to monitoring air pollution. Despite the challenges, the group members recognized the importance of air pollution to health and the need to study its effects across the lifespan to further understand the cumulative effects of different pollutants. There was a specific call to action for the use of effective and affordable monitors at a macro (ambient air) and local (indoor cooking exposure and cooking over open fires) level. Such monitors could then be deployed widely for data collection. 

### Biomarkers of chemical exposure and infectious disease

There are major gaps in infectious disease and environmental exposure research, which represent major opportunities for this field. The impact of chemical exposures on the immune system and susceptibility to infection or progression in infectious diseases has been minimally explored in primary research settings. Exposures considered relevant to infectious diseases included: air pollution (traffic/car fumes/particulate matter, cooking and heating with coal, and indoor air pollution), mining, naturally occurring food toxicants and contaminants, pesticides, insecticides, heavy metals, and high voltage power lines. There was also a general interest in the genetics of susceptibility to environmental exposures. Issues of temporality may impact our understanding of how environmental exposures impact infectious diseases. For example, exposures may impair the immune system and make the body more susceptible to infections. Infections may also lead to immunocompromised states and greater susceptibility to the harmful effects of environmental exposures. Disentangling the cause and effect of infection, immunity, environmental exposures, and disease susceptibility was noted as an important consideration for future research, particularly in the context of a well characterized genome.

## Panel discussion

Panelists included Dr. Mark Nicol, from University of Cape Town, Dr. Michèle Ramsay from the University of the Witwatersrand, Dr. Kiros Berhane from University of Southern California, Dr. Brenda Eskenazi, University of California Berkeley, and Dr. Adrie Steyn from the Africa Health Research Institute and University of Alabama Birmingham.

Some additional general considerations of study design, chemical properties of exposures (half-life), sample types available in study populations, timing of sample collection, variability in exposure levels across individuals, and the complexity of multiple exposures were mentioned in the panel discussion. There was a lengthy dialogue about whether an agnostic approach vs. hypothesis-driven approach was more useful for beginning to explore the impact of different environmental exposures on health outcomes in Africa. Some panelists and the audience felt that targeted hypothesis-directed research would be most effective. This would require further exploration of what might be feasible within existing H3Africa studies. Others felt that an exploratory hypothesis-free approach would be more useful, given that little is known about the range and diversity of exposures present in many African populations. Dr. Nicol advocated for a cross-H3Africa Consortium study of biomarkers of exposure by exploring the possibility of using existing archived specimens to generate an initial broad assessment of relevant exposures using key biomarkers. This might further inform research priorities and assist in developing hypotheses. One challenge with this approach is the selection of biomarkers and the accuracy they would offer as proxies for specific exposures, especially given that the protocols for sample collection varied across studies.

There was additional discussion on leveraging prospective H3Africa studies and identifying opportunities for collection of biospecimens. Given that some H3Africa studies were planning for sample collection, there is potential to harmonize the collection, storage, and sampling to assess potential environmental exposures across studies. Investigators could potentially collaborate with other NIEHS-funded investigators and expand their work in existing study populations by collecting environmental data, analyzing stored biospecimens, planning the collection of new biospecimens, or integrating biomonitoring data (e.g. from air monitors or satellites) into their studies. A variety of potential collaborative opportunities or joint publications relating to common environmental exposures, life stages, and health outcomes across different H3Africa study populations might be possible. Some discussion focused on developing and distributing standardized environmental exposure questionnaires. Phenotypes and Exposures ( PhenX) toolkit and other existing questionnaires were discussed. Dr. Eskenazi mentioned a short questionnaire developed by the International Fetal and Newborn Growth Consortium, which could be used as an example and potentially tailored to African populations. This questionnaire was shared with H3Africa environmental health working group members after the workshop and has been previously described ^33^. 

The ability to leverage existing expertise to explore the measurement of environmental exposures in different samples was also a key focus of the panel discussion. The NIEHS CHEAR ^31^, which is transitioning to NIEHS HHEAR (Human Health Exposure Analysis Resource), was mentioned as a mechanism to measure more environmental exposures in existing H3Africa or related study populations. Current H3Africa investigators could use CHEAR/HHEAR to explore more environmental exposures in their populations and more broadly study gene-environment (G x E) of complex disease in African populations.

## Poster presentations

Fifteen poster presentations by trainees covered environmentally focused research efforts that emphasized some of the unique challenges for this field in Africa. Topics highlighted in the posters included: indoor and outdoor air pollution, coal mining exposures, respiratory microbiota, and pesticide exposures. Research presented by these trainees covered topics such as multidrug-resistant *Plesiomonas shigelloides* in surface waters in Southwest Nigeria, traffic-related air pollution exposure and health outcomes in street vendors in Cape Coast, Ghana, PM2.5 concentrations in Kampala, Uganda, household air pollution and stroke in Ghana and Nigeria, inhaled air pollution and the microbiota of children in the Gambia, pesticide residues in fruits and vegetables in Kampala, Uganda, and characterization of PM2.5 in Addis Ababa. Abstracts for all posters are included in the workshop meeting book posted on the workshop website.

## Next steps: H3Africa Environmental Health Working Group

To address the environmental research needs of the H3Africa Consortium, a new H3Africa working group was initiated in 2018, referred to as the H3Africa Environmental Health Working Group. In the coming years, this working group hopes to continue to build on the momentum from the workshop to explore ideas of individual and collaborative projects facilitating the incorporation of environmental risk factors into H3Africa studies. These may include identification of potential collaborative opportunities or joint publications relating to common environmental exposures across different H3Africa study populations. This group will investigate ways to strengthen capacity to measure environmental exposures and expand research in environmental epidemiology, including research on the epigenome and microbiome, and related G x E mechanisms. The group will also discuss ways investigators can expand their work in existing study populations by collecting environmental data through questionnaires, analyzing stored biospecimens for biomarkers of exposures, planning the collection of new biospecimens, or integrating data from air and personal monitors as well as satellite-based environmental data. Attention to the current issues of environmental health research such as mixtures, where multiple environmental/chemical exposures are measured in a sample/individual is also important. Education on the most appropriate statistical methodology for analyzing mixtures data, rather than traditional epidemiology models, has been discussed and ongoing training through webinars, other virtual methods, and in-person meetings is anticipated.

## Future directions

With developments in technologies to measure environmental exposures, there is opportunity for global progress in environmental health research. In Africa, important environmental research has enabled interventions to reduce harmful exposures (e.g., aflatoxin). However, much more could be addressed. One of the most pressing concerns identified through this workshop was the underlying need for further characterization of key environmental exposures across varying geographies and populations in Africa. Against the backdrop of challenges of malnutrition, infectious diseases, and limited environmental regulations, the environmental exposure burden of Africans appears to be high, and likely contributes substantially to disease. Importantly, the prevalence of cardiovascular disease, obesity, and diabetes is increasing in African populations, particularly in some geographic regions such as southern Africa, impacting both children and adults ^34^. Leadership by African researchers on studies evaluating multiple environmental exposures, gene-environment interactions, and comparative biology is important and timely. The H3Africa Consortium, with accomplishments in genomic research infrastructure, trained staff, research productivity, and extensive clinical and demographic data across African study populations, represents an ideal platform for advancing African-led environmental health science across the continent. Considering that the environment is a modifiable risk factor, this research has viable application to important intervention and prevention efforts.

## Disclaimer

The views expressed in this article are those of the authors and are not meant to be representative of the affiliated institutions. Publication in AAS Open Research does not imply endorsement by the AAS.

## Data availability

Data relating to the survey completed by nine projects prior to the workshop is available in Table 1.
