# Supplementary material for: Integrating environmental health and genomics research in Africa: challenges and opportunities identified during a Human Heredity and Health in Africa (H3Africa) Consortium workshop
Source: AAS Open Res. 2019 Aug 27;2:159. [Version 1] doi: 10.12688/aasopenres.12983.1 (PMC7194141; doi:10.12688/aasopenres.12983.1)
Supplement: Peer review history

# Peer review: Reviewer response for version 1

**DOI:** 10.21956/aasopenres.14064.r27162

**Published:** 2019-10-15

**Authors:** Simon M. Agwale

**Affiliations:** 1 Innovative Biotech, Keffi, Nigeria

This is a compelling letter by Joubert *et al.* The article discusses the outcome of a workshop on environmental health research in Africa that was organised by the NIEHS in conjunction with the H3Africa Consortium and African Society of Human Genetics. The authors highlight progress made by the H3Africa Consortium and provide justification for more education, training, and research on environmental health to be incorporated within the H3Africa network. Moreover, presentations during the workshop are summarized, the need to leverage on H3Africa network and integrate genomics and environmental health research is discussed. It was thoughtful that during the workshop, H3Africa Environmental Working Group was initiated to explore ways of incorporating environmental risk factors into studies conducted by the H3Africa going forward. Overall, this is an engaging manuscript that highlights the appreciable progress made by the H3Africa Consortium and emphasizes the need to integrate environmental health and genomics research in Africa within the H3Africa studies.

I confirm that I have read this submission and believe that I have an appropriate level of expertise to confirm that it is of an acceptable scientific standard.

# Response

**Published:** 2019-10-17

**Authors:** Bonnie Joubert

**Affiliations:** NIEHS, USA

We thank the reviewer for these positive comments.

# Peer review: Reviewer response for version 1

**DOI:** 10.21956/aasopenres.14064.r27165

**Published:** 2019-09-24

**Authors:** Riana M.S. Bornman

**Affiliations:** 1 UP Institute for Sustainable Malaria Control, University of Pretoria, Pretoria, South Africa

**Approval status – ‘Approved’: **One minor change is required.

**Review:**

- The rationale for this Open Letter was clearly motivated in sufficient detail. The factual statements are correct and adequately supported by citations. The paper also discusses differing views and opinions, where applicable.
- The Letter is well written in accessible language. The detailed recommendations and next steps provide valuable direction for others to follow.
- The inclusion of Student Posters in the Letter is highly recommended.
- This paper also provides a key summary of most published information on environmental data and interests in H3Africa projects. It will be a valuable reference for future use for researchers from Africa.

**Please check**:

- Page 8 – The header uses “Pesticides” in terms of IRS and it should be “Insecticides”.

I confirm that I have read this submission and believe that I have an appropriate level of expertise to confirm that it is of an acceptable scientific standard.

# Response

**Published:** 2019-10-09

**Authors:** Bonnie Joubert

**Affiliations:** NIEHS, USA

Thank you for the careful review of this manuscript. We agree with the reviewer comments. We will clarify the term pesticides to insecticides when referring specifically to indoor residual spraying (IRS).
